# Chlorinated persistent organic pollutants in human breast milk in the Miyagi Prefecture disaster-affected area 1 year after the Great East Japan Earthquake of 2011

**DOI:** 10.1265/ehpm.22-00260

**Published:** 2023-05-02

**Authors:** Jungmi Choi, Yukiko Fujii, Zhaoqing Lyu, Hatasu Kobayashi, Tomoko Fujitani, Kouji H. Harada

**Affiliations:** 1 Department of Health and Environmental Science, Kyoto University Graduate School of Medicine, Yoshida konoe, Sakyo, Kyoto 606-8501, Japan; 2 Department of Research Promotion and Management, National Cerebral and Cardiovascular Center Research Institute, 6-1 Kishibe-Shimmachi, Suita, Osaka 564-8565, Japan; 3 Department of Pharmaceutical Sciences, Daiichi University of Pharmacy, 22-1 Tamagawa-machi, Minami-ku, Fukuoka 815-8511, Japan; 4 Department of Environmental and Molecular Medicine, Mie University Graduate School of Medicine, 2-174 Edobashi Tsu, Mie 514-8507, Japan

**Keywords:** Persistent organic pollutant, Breast milk, Japan, Great East Japan Earthquake, Natural disaster

## Abstract

**Background:**

In the Great East Japan Earthquake of 11 March 2011, an earthquake and accompanying tsunami struck the Tohoku region of northeastern Japan. Buildings collapsed and the tsunami spread waste, including hazardous materials. This study aimed to determine the concentrations of persistent organic pollutants (POPs) in the breast milk of mothers living in the disaster-affected area of Sendai 1 year after the earthquake. Temporal trends in the POPs concentrations were evaluated by comparison with previous studies.

**Methods:**

One hundred breast milk samples were obtained from lactating mothers at a hospital in Sendai in 2012. The results were compared with those from other years to examine whether there were changes in the POPs concentrations after the earthquake. We measured polychlorinated biphenyls (PCBs) and organochlorine pesticides, such as chlordanes, using gas chromatography-mass spectrometer (GC-MS) with negative chemical ionization, and dichlorodiphenyl trichloroethane (DDT) and its metabolites using GC-MS with electron impact ionization.

**Results:**

The mean total PCBs (11 congeners), total chlordane, and total DDT concentrations were 76.2 ng/g lipid, 39.8 ng/g lipid, and 73.5 ng/g lipid, respectively. For the samples collected in 2012, the concentrations of POPs in breast milk showed minimal changes compared with results from previous years for samples collected at the same hospital in Sendai.

**Conclusions:**

Our study demonstrates that 1 year after the earthquake and tsunami, the concentrations of chlorinated POPs in breast milk had not changed substantially.

## Background

In the Great East Japan Earthquake of 11 March 2011, a magnitude 9.0 earthquake and accompanying tsunami struck the Tohoku region of northeastern Japan. Tsunami waves reached a height of up to 41 m and caused extensive damage [1]. The tsunami and earthquake caused extreme human suffering and destroyed homes and industrial facilities in the coastal areas. The health effects of the disaster persisted after several years [2–4]. Furthermore, many radionuclides were leaked into the environment because of an accident at the Fukushima Daiichi Nuclear Power Plant that accompanied the disaster [5, 6]. In addition to radionuclides, tsunami-damaged industrial facilities leaked many chemical substances into the environment [7, 8]. Environmental contamination by toxic pollutants after this disaster is of concern [9].

Around the world, several environmental surveys have been conducted for naturally occurring and man-made chemicals in tsunami deposits after disasters [10, 11]. In many cases, the excess concentrations of pollutants such as lead and arsenic over the soil environmental standard values in tsunami deposits could originate from the seafloor/river sediment and seawater. However, locally significant contamination with fluorine and oil has been observed [10], and these pollutants may originate from damaged industrial buildings.

A tsunami could result in persistent organic pollutants (POPs), such as polychlorinated biphenyls (PCBs), leaking into the environment from storage sites [12]. Even small amounts of POPs in the environment may cause negative effects because they can accumulate in high concentrations in the bodies of higher-order predators. Several studies have investigated the concentrations of toxic chemicals in the marine environment, including some POPs in mussels and insects, after the 2011 disaster in Japan [13, 14]. Interestingly, PCB levels in mussels have increased significantly in affected areas [15]. These changes might affect the human exposure to POPs and other chemicals via seafood consumption. Except for radioactive substances, however, few exposure assessments have investigated chemical substances in human samples such as breast milk or serum.

In this study, we focused on POPs that could accumulate in humans and that pose a risk to human health after the 2011 earthquake. To evaluate the human exposure risk 1 year after the earthquake, we analyzed the levels of POPs in breast milk samples from Sendai (prefectural capital city of Miyagi Prefecture, Tohoku region, Japan) in 2012. Temporal trends of POPs concentrations were evaluated by comparison with the results from previous studies [16–18].

## Methods

### Sample collection

Breast milk samples from 2012 were sourced from Sendai, Miyagi Prefecture. Samples from this region have been continuously banked in the Kyoto Human Specimen Bank since 2005 for chemical analysis of environmental pollutants [19–21]. Samples were obtained from lactating mothers within 2 months of delivery. The mothers gave birth at a general hospital in Sendai between June and December of 2012. The donated average mass of breast milk per person was 326 g (range: 93–400 g). The information on age, the number of births, smoking, and drinking statuses was collected via a self-report questionnaire. The hospital recorded 1229 deliveries in 2012. The protocol for this study was approved by the Ethics Committee of the Graduate School of Medicine, Faculty of Medicine, Kyoto University, and the Faculty of Medicine Hospital (R1478). Written consent was obtained from all breast milk donors.

### Chemical analysis

PCBs (CB118, CB138, CB146, CB153, CB156, CB170, CB180, CB182/187, CB194, CB199, and CB206), hexachlorocyclohexanes (α-HCH, β-HCH, and γ-HCH), chlordanes (*cis*-CHL, *trans*-CHL, oxychlordane, *cis*-nonachlor, and *trans*-nonachlor), pentachlorobenzene (PeCB), hexachlorobenzene (HCB), heptachlor epoxide, toxaphenes (Parlar #26 and #50), and dichlorodiphenyltrichloroethane (DDT) and its metabolites (*p*,*p*′-DDT, *p*,*p*′-DDE, and *p*,*p*′-DDD) were analyzed as the target chemicals. Expanded POPs Pesticides Calibration Solutions CS1–CS6 (ES-5464), Expanded POPs Pesticides Cleanup Spike (ES-5465), POPs Toxaphene Calibration Solutions with PCB Syringe (ES-5351), and Expanded POPs Pesticides Cleanup Spike (ES-5465), used as standard solutions for quantification and were purchased from Cambridge Isotope Laboratories (Tewksbury, MA, USA). Native PCB Solution/Mixture for MS Detection (BP-MS) and Mass-Labelled PCB Congeners (P48-M-ES) were purchased from Wellington Laboratories (Guelph, Ontario, Canada). We used ^13^C_12_-2,3,3′,5,5′-pentachlorobiphenyl (CB-111, Cambridge Isotope Laboratories) as an internal standard for quantification. Isopropanol, diethyl ether, hexane, nonane, and dichloromethane were used for residual pesticide tests and PCB tests (Kanto Chemical Co., Inc., Tokyo, Japan).

Before analysis, each breast milk sample was stirred, and then a 5-mL aliquot was placed in a polypropylene centrifuge tube. Next, 9 mL of extraction solvent (isopropanol/diethyl ether/hexane, 2:1:3 v/v/v) and 500 pg of ^13^C-labeled standards (PCBs, organochlorine pesticides) were added. After vortex mixing, the mixture was centrifuged (1570 × *g*). The organic layer was transferred to a flask and the extraction was repeated with 8 mL of extraction solvent. The organic layers from the two extractions were combined and concentrated using a rotary evaporator. The crude extract was diluted to a total volume of 10 mL with hexane. Some of the sample was removed at this stage to determine the lipid content. Distilled water was added to the remaining crude extract and it was vortex mixed, centrifuged (1570 × *g*), and the water layer was removed. Next, about 10 mL of the remaining extract was added to a column containing 2 g of activated Silica gel (Wako Pure Chemicals, Osaka, Japan) and 6 g of activated Florisil (Wako Pure Chemicals, Osaka, Japan) and eluted with 20 mL of hexane (first fraction) and 40 mL of a 10% dichloromethane/hexane solution (second fraction). The eluate was concentrated to approximately 1 mL using a rotary evaporator. Nonane was added to the concentrate and the sample was further concentrated to 0.1 mL. Finally, ^13^C_12_-labelled CB-111 was added for gas chromatography-mass spectrometry (GC-MS) analysis.

A GC-MS (Agilent 6890/5973i, Agilent Technologies, Santa Clara, CA, USA) with a capillary column (HP-5MS, 30 m × 0.25 mm i.d., 0.25 µm film thickness, Agilent Technologies) was used for analysis. DDTs were analyzed using electron impact ionization. Other substances were analyzed by negative chemical ionization using methane gas. The instrument detection limit was defined as a signal-to-noise ratio of three. The method detection limit was assumed to be equal to the instrument detection limit because it was below the instrument detection limit for blank samples. A blank sample was analyzed after every 10 samples to assess if contamination occurred during the extraction and purification steps.

## Results and discussion

### Study participants

Breast milk samples were obtained from 100 donors. Characteristics of donors, such as the age, the number of births, smoking, and drinking statuses are listed in Table 1. These characteristics of the donors were comparable to those in a nationwide survey [22, 23].

**Table 1:** Characteristics of donors of breast milk samples in Sendai in 2012 (*n* = 100)

Age (years)	mean ± SD	31.5 ± 5.0
	range	20–42

		n (%)

Parity (*n*)	1	58 (58)
	2	29 (29)
	3	8 (8)
	4	3 (3)
	Missing	2 (2)

Smoking (*n*)^a)^	Non	48 (48)
	Ex	18 (18)
	Current	1 (1)
	Missing	33 (33)

Drinking (*n*)^a)^	Non	29 (29)
	Ex	56 (56)
	Current	0 (0)
	Social^b)^	12 (12)
	Missing	3 (3)

a) Status at time of collection of breastmilk.

b) Drinking alcohol only in social settings, such as social gatherings and reception.

### Overall trends and changes in POPs before and after the earthquake

In this study, we measured 11 PCB congeners and eight pesticides (17 isomers/metabolites) in breast milk samples (Table 2).

**Table 2:** Mean POPs concentrations in breast milk samples (*n* = 100) from Sendai, Japan in 2012

**Compound**	**% of detection**	**Concentration ** **(ng/g lipid)**
CB118	79	1.31 ± 1.37
CB146	100	3.88 ± 2.86
CB153	100	26.2 ± 18.5
CB138	100	18.1 ± 13.3
CB156	89	0.83 ± 0.71
CB182/187	99	6.56 ± 4.73
CB180	100	11.8 ± 8.31
CB170	100	3.90 ± 2.68
CB199	100	1.31 ± 0.85
CB194	99	1.03 ± 0.65
CB206	100	0.28 ± 0.55
ΣPCB	-	76.2
α-HCH	70	0.20 ± 0.31
β-HCH	100	56.2 ± 43.6
γ-HCH	35	0.67 ± 0.77
ΣHCH	-	57.1
PeCB	95	0.55 ± 0.49
HCB	100	11.6 ± 7.40

oxychlordane	100	10.8 ± 9.74
*trans*-CHL	99	0.46 ± 1.35
*cis*-CHL	99	0.44 ± 0.61
*trans*-nonachlor	100	24.7 ± 24.3
*cis*-nonachlor	100	3.37 ± 3.21
ΣChlordane	-	39.8
Heptachlor epoxide	100	4.20 ± 4.57
Mirex	97	1.31 ± 0.99

Toxaphene (Parlar 26)	38	1.52 ± 5.33
Toxaphene (Parlar 50)	59	1.64 ± 6.34
ΣToxaphene	-	3.17
*p*,*p*′-DDE	100	69.6 ± 88.3
*p*,*p*′-DDT	74	2.24 ± 2.65
*p*,*p*′-DDD	59	1.66 ± 2.12
ΣDDT	-	73.5

Data are presented as means ± standard deviations.

To evaluate temporal changes after the 2011 earthquake, the concentrations of the target compounds were compared with those from other studies for samples from the same hospital in Sendai in 2005, 2007, and 2009 [16–18] (Table 3).

**Table 3:** POPs concentrations in breast milk samples from the same hospital in Sendai in different years.

**Compound**	**Concentration (ng/g lipid)** **Sampling year (number of participants)^a^** **Reference**			
	**2005 (*n* = 40)**	**2007 (*n* = 20)**	**2009 (*n* = 30)**	**2012 (*n* = 100)**
	**Inoue et al. [18]**	**Haraguchi et al. [17]**	**Fujii et al. [16]**	**Present study**
ΣPCB	78.5 (40.7)	150 (69–360)	129	76.2 (51.4)
α-HCH	n.a.	n.a.	0.26	0.20 (0.31)
β-HCH	n.a.	n.a.	89	56.2 (43.6)
γ-HCH	n.a.	n.a.	0.11	0.67 (0.77)
HCB	n.a.	18 (6.4–31)	19	11.6 (7.40)
ΣChlordane	n.a.	47 (8.6–140)	58	39.8 (35.7)
Heptachlor epoxide	n.a.	n.a.	5.1	4.20 (4.57)
Mirex	n.a.	n.a.	1.1	1.31 (0.99)
Toxaphene (Parlar 26)	n.a.	n.a.	1.1	1.52 (5.33)
Toxaphene (Parlar 50)	n.a.	n.a.	2.1	1.64 (6.34)
ΣDDT	n.a.	260 (65–970)	n.a.	73.5 (90.1)

Data are presented as means with the standard deviation or the range in parentheses. The abbreviation n.a. indicates that samples were not analyzed for the target chemical in that year.

^a^Data were obtained from Inoue et al. [18] for 2005, Haraguchi et al. [17] for 2007, Fujii et al. [16] for 2009, and the present study for 2012. For the 2009 data, the 30 samples were pooled to form 10 sets of three samples.

The total PCBs concentration (11 congeners) range was 15.2–242 ng/g lipid (mean: 76.2 ng/g lipid), and was similar to the concentrations measured in previous studies (78.5 ng/g lipid in 2005 [18], 150 ng/g lipid in 2007 [17], and 129 ng/g lipid in 2009 [16]). The pattern of PCB homolog and isomer was also consistent with previous studies [16–18]. The main HCH was β-HCH, which accounted for 98% of the total HCHs. The β-HCH concentration range was 8.90–196 ng/g lipid (mean: 56.2 ng/g lipid), and these concentrations were slightly lower than those measured in 2009 (mean: 89 ng/g lipid) [16]. The HCB concentration range was 2.78–58.9 ng/g lipid (mean: 11.6 ng/g lipid), which was consistent with the concentrations measured in 2007 (18 ng/g lipid) [17] and 2009 (19 ng/g lipid) [16].

The concentration range for PeCB, which has never been measured in Japan but was designated as a POP in the Stockholm Convention in 2009, was 0.05–3.45 ng/g lipid (mean: 0.55 ng/g lipid). Compared with the other POPs, PeCB was present at trace concentrations. Additionally, the PeCB concentrations were similar to those measured in a study in Denmark [24]. The mean total chlordane concentration in breast milk was 39.8 ng/g lipid. Technical chlordane contains heptachlor, *trans*-CHL, *cis*-nonachlor, and *trans*-nonachlor. Chlordanes are converted to the metabolite, oxychlordane *in vivo*, and heptachlor is converted to heptachlor epoxide in soil and *in vivo*. In the breast milk samples, we found that *trans*-nonachlor and oxychlordane were the main chlordanes, which was consistent with the results from previous studies [16, 17]. The mean total chlordane concentration measured in 2007 was 47 ng/g lipid [17] and that measured in 2009 was 58 ng/g lipid [16]. These concentrations were similar to those measured in the present study (39.8 ng/g lipid). Toxaphene (Parlar 26 and Parlar 50) and mirex were detected in the breast milk samples, although they are not registered as pesticides in Japan. Toxaphene has been detected in the Japanese diet [25] and this may be from imported food. In fact, toxaphene was used in the neighboring countries, such as Republic of Korea until 1999 [26] and People's Republic of China until 1981 [27]. On the other hands, mirex was never registered as a pesticide in Japan but was used as a flame retardant (named dechlorane) [28]. Detection of mirex in breast milk of Japanese residents presumably results from exposure of dechlorane through air and food.

The mirex concentration range was 0.20–6.32 ng/g lipid (mean: 1.31 ng/g lipid), and the total toxaphene concentration range was 0.39–350 ng/g lipid (mean: 3.17 ng/g lipid). The concentrations of the isomers Parlar 26 and Parlar 50 were high. The average concentration was not substantially different from that measured in 2009 [16]. The total concentration range of DDTs (*p*,*p*′-DDT, *p*,*p*′-DDE, and *p*,*p*′-DDD) in breast milk was 3.28–670 ng/g lipid (mean: 73.5 ng/g lipid). This mean concentration was lower than that for the total DDT concentrations in 2007 (260 ng/g lipid). Among DDTs, *p*,*p*-DDE was the main constituent (95% of the total concentration).

In summary, our results show that the concentrations of POPs in breast milk had not substantially changed after the tsunami compared with those before the tsunami.

### Limitations of this study

There were some limitations. First, subjects in this study were not representative samples in Miyagi Prefecture. However, at least, these were case series in different years at a single center, and it may explain the temporal change in POPs exposure among the selected population in this study. Second, the number of samples was relatively small in this study, particularly in previous comparative studies (40 samples; year 2005 [18], 30 samples; year 2009 [16], and 20 samples; year 2007 [17]). Since POPs concentration in breast milk has high individual variability and often shows the abnormal distribution, the sample size could be a potential limitation. Moreover, the lack of detailed adjustment of the background information on participants, such as age, is a limitation of this study and may have affected the findings. Third, breast milk samples were collected only 1 year after the disaster. At this stage, human exposure to POPs because of the disaster might not be reflected in the POPs concentrations in breast milk. Although, high concentrations of PCBs and polybrominated diphenyl ether in lower trophic level fish species have been observed in the disaster-affected area [29], human exposure to these chemicals over time will be gradual, and this will be reflected in the concentrations in human biological samples. There is a precedent for long-term studies to monitor chemical contamination of abiotic samples after an earthquake, such as in the Bay of Concepción, Chile [30]. Moreover, the disaster may have temporarily changed people’s behaviour, for example, by discouraging the consumption of fish for fear of radionuclide contamination after the nuclear disaster [31]. Therefore, we suggest that continuous monitoring is also required in the Tohoku region of northeastern Japan.

## Conclusions

One year after the earthquake and tsunami, the concentrations of chlorinated POPs in breast milk samples in Miyagi Prefecture had not changed substantially.

## Data Availability

The data that support the findings of this study are available from the corresponding author upon reasonable request.

## Abbreviations

POPs: persistent organic pollutants
PCBs: polychlorinated biphenyls
HCH: hexachlorocyclohexanes
CHL: chlordanes
PeCB: pentachlorobenzene
HCB: hexachlorobenzene
DDT: dichlorodiphenyltrichloroethane
GC-MS: gas chromatography-mass spectrometry
